# Developing a deprivation index to study geographical health inequalities in Ecuador

**DOI:** 10.11606/s1518-8787.2019053001410

**Published:** 2019-11-21

**Authors:** Andrés Peralta, Verónica Espinel-Flores, Mercè Gotsens, Glòria Pérez, Joan Benach, Marc Marí-Dell’Olmo

**Affiliations:** I Agència de Salut Pública de Barcelona. Barcelona, Catalonia, Spain; II Universitat Pompeu Fabra. Department of Political and Social Sciences. Health Inequalities Research Group, Employment Conditions Knowledge Network (GREDS-EMCONET). Barcelona, Spain; III Johns Hopkins University - Pompeu Fabra University Public Policy Center, Barcelona, Spain; IV Universitat Pompeu Fabra. Department of Experimental and Health Sciences. Barcelona, Catalonia, Spain; V Institut d’Investigació Biomèdica (IIB Sant Pau). Barcelona, Spain; VI CIBER Epidemiología y Salud Pública (CIBERESP), Spain; VII Universidad Autónoma Madrid. Transdisciplinary Research Group on Socioecological Transitions (GinTRANS2). Madrid, Spain

**Keywords:** Social Determinants of Health, Social Inequity, Poverty, Mortality, Ecuador

## Abstract

**OBJECTIVES:**

To develop a deprivation index to study health inequalities in 221 areas of Ecuador, to describe the pattern of deprivation in Ecuador, and to explore the applications of the index to study health inequalities by analysing the association between deprivation and mortality in the study areas.

**METHODS:**

We performed principal component analyses of available indicators of the 221 cantons of Ecuador. A set of 41 sociodemographic, social capital, and subjective well-being variables were obtained from the 2010 National Population Census and the National Living Conditions Survey 2013–2014. To explore the application of the index in public health, the association between the index and standardised mortality ratios was estimated through a Poisson regression model.

**RESULTS:**

The final index was constructed with 17 indicators. The first component explained 51.8% of the total variance of the data. A geographic pattern and a positive association of the index with the standardised mortality ratios of the cantons were observed in both men and women.

**CONCLUSIONS:**

We constructed a deprivation index that can identify disadvantaged areas in Ecuador. This index could be a valuable tool for the detection of vulnerabilised populations and the development of interventions and policies adapted to local needs.

## INTRODUCTION

Although extreme poverty has been substantially reduced in Ecuador in the last decade, geographical, ethnic and gender-based inequalities persist in the country^1^. As many other Latin American countries, Ecuador inherited a colonialist model grounded in patriarchy, classism and racism that have created a social stratification benefiting social elites that continue to exert strong control over land tenure and wealth^1^. Consequently, women, peasant communities, indigenous people and Afro-Ecuadorian groups are living in poverty as a collective experience of structural and historical origin^2^. Regarding the structural complexity resulting from power relationships, intersectionality analysts and theorists have argued that “inequities are never the result of single, distinct factors. Rather, they are the outcome of intersections of different social locations, power relations and experiences”^3^.

Power relationships also determine the distribution of material and social resources needed by individuals to achieve the living standards considered essential by their social and cultural groups. The lack of these resources has been defined in the literature as material and social deprivation^4^. Deprivation is usually considered a collective experience and therefore is often measured in public health and epidemiology as a contextual phenomenon. These measurements have been widely used to describe the social determination of health and the impact of the context on the health of populations. Deprivation is a complex construct with multiple dimensions that can change across contexts and periods. Nevertheless, most efforts to conceptualise and measure material and social deprivation have focused on high-income countries^5^. Few studies have analysed how multiple types of deprivation shape people’s health and health inequities in middle- and low-income countries^6^.

Health is recognised as being essential to achieve key objectives in life such as educational attainment and adequate employment conditions, and to promote the freedom of individuals and societies^7^. Furthermore, health capability provides individuals and communities with the “opportunity to achieve good health and thus be free from escapable morbidity and mortality”^8^. Therefore, assessing the health status of a population and its distribution is an aim of political and societal activity of crucial ethical importance to achieve social justice. If deprivation affects health, its measurement is therefore of vital importance to reduce social inequalities and promote social justice.

Deprivation can be measured with simple indicators or indices constructed by merging indicators representing one or multiple dimensions. Indices are used when a single indicator or measurement is insufficient to describe a complex phenomenon such as deprivation^9^. The measurement of deprivation in geographical areas and its relationships with health over time has proven to be useful for health surveillance, inequality analysis, resource allocation, and priority setting^5^^,^^9^. In Ecuador, prior attempts at measuring deprivation^1^^,^^2^^,^^10^^,^^11^ present methodological limitations for the study of health inequalities related to: i) lack of representativeness at smaller geographical units; ii) estimations made in non-comparable geographical units (different administrative levels for urban and rural estimates); and iii) inclusion of limited core deprivation dimensions and indicators. A deprivation index specifically designed for the study of health inequalities has, to the extent of our knowledge, never been constructed in Ecuador.

Therefore, we sought to develop a deprivation index for the study of health inequalities in 221 cantons of Ecuador (the second admistrative level) to describe the geographical pattern of deprivation and to explore an application of the index for the study of health inequalities by analysing the association between deprivation and mortality in the study areas.

## METHODS

### Study Design and Information Sources

**Supplementary Figure 1:**
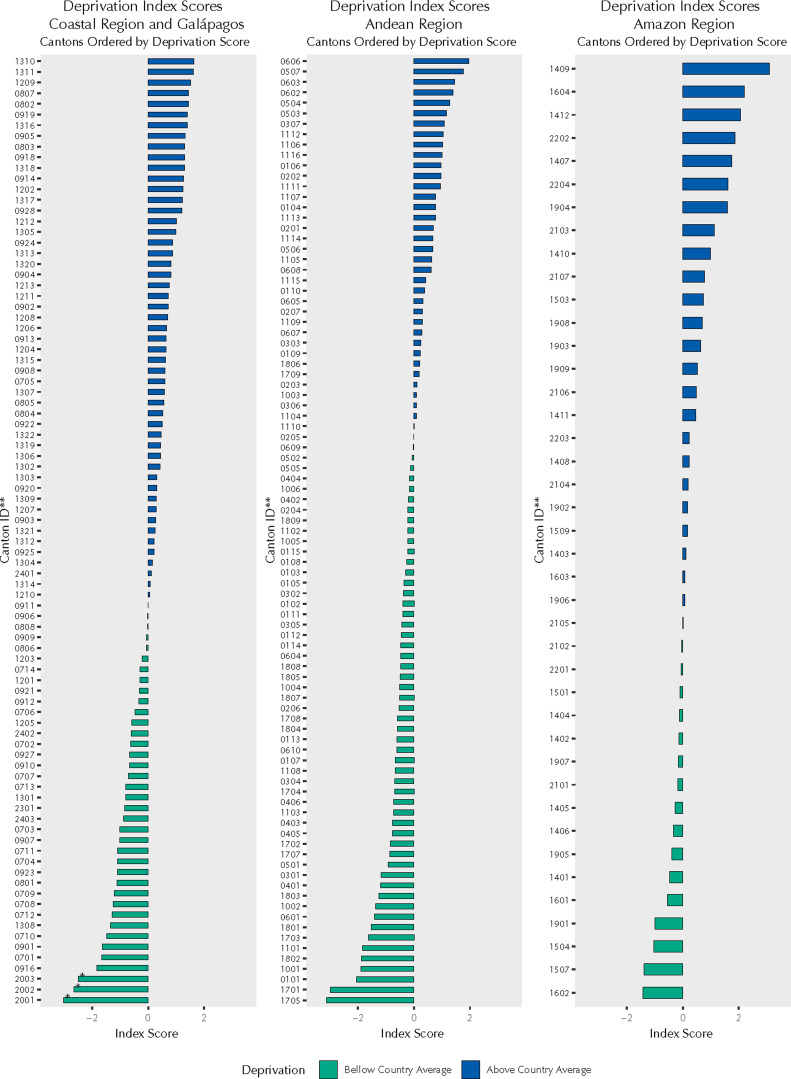
Geographical distribution of the 221 cantons of Ecuador by geographical regions.

An ecological study was performed using the 221 cantons of Ecuador as units of analysis, according to the last National Population Census 2010. Cantons were selected as area of study for two reasons: i) Cantons are the smallest administrative level with comparable data available. Each canton has a unique history, social and natural environments, and political representation; and ii) Cantons are the smallest administrative level with the capacity to design and implement local policies. Each canton has its own political representation, an elected mayor and local elected council members; and budget. People living in a canton share a history of common local policies that can affect wealth distribution. Canton size and population present important variations. In 2010, the population of the cantons ranged from 1,760 to 2,350,278 (median 23,820) inhabitants. These 221 areas are geographically located in four different regions (Supplementary Figure 1): 1) The Galápagos Islands in the Pacific Ocean; 2) The coastal region in the western part of the continental territory; 3) the Amazon region in the eastern portion of the continental territory; and 4) the Andean region in between. Demographic and socioeconomic data were obtained from The National Population Census 2010. Social capital and subjective well-being measures were estimated from the national Living Conditions Survey 2013–2014. Data from the LCS are representative at the provincial level, the first administrative level. Provincial indicators from this source were assigned to all the cantons composing each province. Mortality data were obtained from the Ecuadorian mortality registry for 2009 to 2011. All databases were obtained from Ecuador´s National Institute of Statistics and Censuses (INEC) and are available online (http://www.ecuadorencifras.gob.ec/estadisticas/).

### Definition of Deprivation, its Dimensions in the Ecuadorian Context and Selection of Variables

For this study, we first created a definition of deprivation considering the context. This definition was constructed after a literature review that included relevant documents from the Latin American Social Medicine movement^12^, official documents from the country, e.g. development plans and the national constitution; and conceptual discussions between the authors. Deprivation was defined as a historically and structurally determined collective phenomenon linked to human rights violations; this creates a social stratification in the country based on power structures and relations that privilege certain social groups by oppressing and exploiting others. This stratification affects the capabilities of certain population segments to achieve decent living standards, which also affects their health.

Subsequentially, we performed a comprehensive search for available data in the country to operationalise our definition of deprivation. Then indicators were distributed in seven deprivation dimensions: 1) Education, 2) Information and Communication, 3) Housing and Urban Environment, 4) Work and Social Security, 5) No discrimination, 6) Healthy Environment, and 7) Social Environment and Social Capital. These dimensions emerged from a comparison of the literature with the available data. A brief definition of each dimension is provided below:

Education: This dimension can influence a person’s work and income. It can also be related to a person’s ability to acquire the skills and resources necessary in harsh/stressful periods. Therefore, educational deprivation can strongly affect the ability to self-care, health and how people deal with health problems^13^.

Information and Communication: It has been argued that, inequalities in access to the internet and the skills needed for its use among the population may exacerbate existing societal and geographical inequalities based on age, gender, educational level and ethnicity^14^. Due to the increasing dependence on internet-based information, we considered access to and use of the web, mobile phones and computers as assets in our study to measure the digital divide^15^.

Housing and Urban Environment: Secure and adequate housing is essential not only to protect people from environmental conditions, but also to create social bonds and to establish life projects (ontological security). Moreover, housing is interconnected with the surrounding built and social environment (basic services, amenities, community). Housing deprivation and the lack of an adequate urban environment have diverse and substantial health consequences^16^^,^^17^.

Work and Social Security: Paid and unpaid work/labour conditions and the protection given to workers are related not only to income and social stratification, but also to health risks and healthcare access. Income reflects access to material resources and can also reflect social standing, which can affect health through psychosocial processes^13^. Moreover, women’s integration in the paid workforce depends on the societal norms for women’s employment, which can produce multiple problems and ill health by various mechanisms^18^.

Historical Discrimination: Historical oppression and discrimination arising from a post-colonial society can greatly impact living conditions and health. Historically, indigenous and black populations in Ecuador have been oppressed, and politically and economically excluded; while mestizos and especially white populations have enjoyed full participation in the political and economic processes and have had more control over resources and means of production. Therefore, discrimination towards people from minority ethnic groups is an important issue in Ecuador and can intersect with discrimination by gender, health status, age, and rural origin, among others^19^^,^^20^.

Healthy Environment: A pollution-free environment is an important and often neglected component of the quality of life of inhabitants of an area^21^^,^^22^. The Ecuadorian state recognises the importance of the natural environment as a key component of well-being to the extent that it even grants nature basic rights in its Constitution.

Social Environment and Social Capital: Social environment and social support have been proven to be important components of a healthy community and can have considerable health effects^23^. Social deprivation is considered an essential part of multiple deprivation^24^. Thus, variables measuring participation, community work, and quality of relationships with neighbours, and perception of neighbourhood safety were included.

A set of 41 indicators fitting into the above-mentioned deprivation dimensions were selected from our information sources. Indicators were selected or constructed so that higher scores (or percentages) represented less deprivation. The information sources, numerator and denominators of the final variables of the index can be found in the Chart.

**Chart:** Operational definitions of socioeconomic indicators used in the design of a Deprivation Index for Ecuador

Domains/Indicators		Numerator	Denominator	Survey Question
1. Education				
	Literacy^a^.	Literate population	Population of 5 years or more.	19) Can you (...) read and write?
	Educational attainment^a^.	Population with secondary or university education (5–10 years).	Population of 24–65 years.	23) What is the highest level of instruction you attend or attended (...)?
2. Information and Communication				
	Mobile phone access^a^.	Population that made use of a mobile phone in the last 6 months.	Population of 5 years or more.	20) In the last six months (...) have you used: a mobile phone…
	Internet access^a^.	Population that made use of the internet in the last six months.	Population of 5 years or more.	20) In the last six months (...) have you used: the internet…
3. Housing and Urban Environment				
	Main cooking fuel^a^.	Households using adequate fuel for cooking (gas or electricity).	Total of households.	5) Which is the main fuel or energy that this home uses for cooking?
	Toilet for household use only^a^.	Households with exclusive use of a toillet.	Total of households.	3) The household toilet is of: Exclusive use of the household.
	Water Supply^a^.	Dwellings with piped water.	Total of households.	V8) Where does the water received in this home come from?
	Rubbish collection^a^.	Homes that eliminate garbage with a collector car.	Total of households.	V13) Mainly, how do you eliminate the garbage from the home:
	Toilet to sewer.	Households with toilet to sewer.	Total of households.	V9) The household toilet is connected to:
	Electricity supply^a^.	Households with electrical energy (any provider).	Total of households.	V10) The electric light (energy) service of the house comes mainly from:
	Dwellings overcrowding^a^.	Dwellings not overcrowded (3 or less people per bedroom).	Total of households.	14) Without considering the kitchen, bathroom and office, how many bedrooms does the household has? 16) How many persons (households) sleep and cook food?
	Piped into residence/dwelling^a^.	Dwellings with piped water inside.	Total of households.	V8) The water received in this house is:
	Compound indicator of Dwelling’s quality^a^: Walls, Floor, Ceiling	Dwellings in good condition (roof, walls and floors reported as “good”).	Total of households.	V2) The condition of the ceiling or covering of the dwelling is: V04) The condition of the walls of the dwelling is: V6) The condition of the floor of the dwelling is:
4. Work and Social Security				
	Private insurance^a^.	Population with private insurance.	Total of population.	07) (...) has private health insurance?
	Women not payed working hours ratio^b^.	Weekly not payed work hours.	Total worked hours.	24) How many hours a week do you dedicate to the following home chores: (Total hours).
	Poverty perception^b^.	Population that considers not being poor.	Total of households.	4) According to your economic condition, do you consider your home to be:
5. Historical Discrimination				
	Ethnicity^a^.	Population with white or mestizo self-identification	Total of the population.	16) How do you identify (...) according to your culture and customs?

Information Sources:

a Census 2010;

b Life Conditions Survey 2013–2014.

All 17 indicators refer to inhabited dwellings. People residing outside the country when the census was performed were excluded.

### Deprivation Index Construction

As the selected indicators had different measurement units and scales, they were first standardised to obtain new variables, *z*, with mean 0 and variance 1 (by subtracting the area value by the mean of the 221 areas and dividing it by the standard deviation). Next, bivariate Spearman correlations were obtained. When the variables showed correlations greater than 0.90, one of them was removed from subsequent analyses. Those more in agreement with our definition of deprivation were retained.

Sequential principal component analyses (PCA) were performed to maximise the first component variance. PCA can be described as “… a multivariate statistical technique used to reduce the number of variables in a data set into a smaller number of dimensions”^25^. PCA has been widely used to construct socioeconomic and deprivation indexes^9^^,^^26^. The conditions for applying a PCA were assessed using two criteria: 1) Bartlett’s test of Sphericity (statistical significance value set at 5%), and 2) sampling adequacy using the Kaiser-Meyer-Olkin test for the whole dataset and for individual variables. A first PCA was carried out with 36 initial indicators. Then the correlation of each variable with the first component was estimated. Those variables with correlations lower than 0.50 were removed unless they were considered conceptually essential. A final PCA was performed. The final index values are the predicted scores for the 221 areas using the first principal component of the PCA performed with the final variables. To simplify the score interpretation, these values were standardised by subtracting the area value by the mean of the 221 areas and dividing it by the standard deviation to obtain a score z, with mean 0 and variance 1. Finally, the proportion of contribution of each variable to the index was estimated.

A Spearman’s correlation was performed to determine the correlation between an index obtained with the variables of the first PCA (36 variables) and the final index to assess whether a more parsimonious index could be created without losing important information.

The geographical distribution of the deprivation index was displayed using choropleth maps representing the deprivation index scores in five categories (quintiles). To assess the existence of a statistically significant geographical pattern in the index scores (spatial autocorrelation), we used Moran’s *I*^27^. Values of this statistic range from -1 to 1. Significant positive values indicate clustering, significant negative values indicate a regular geographical pattern, and values near 0 indicate a random geographical distribution^28^.

### Use of the Index for Public Health Purposes

We used the population from the 2010 census and all-cause mortality from 2009 to 2011 to assess application and usefulness of the deprivation index for studying socioeconomic inequalities in health in Ecuador. Standardised mortality ratios (SMR) for men and women were estimated using the indirect method, employing the age–specific mortality rates for all Ecuador in the study period as the reference and correcting the number of deaths for mortality completeness estimates. The SMR geographical distribution was displayed using quintile-based choropleth maps. Poisson regression models were performed to estimate the association between the index and SMR for the cantons of Ecuador (for men and women).

All data were analysed using the statistical packages SPSS and R. All maps were plotted using the R statistical package. The R and SPSS codes used for the analyses can be obtained from the corresponding author upon request.

## RESULTS

As shown in Chart, the final deprivation index consisted of 17 indicators (out of the 41 initially selected) describing five different socioeconomic deprivation dimensions. Out of the seven initial dimensions, two (“Healthy Environment”/“Social Environment and Social Capital”) were excluded after conducting the analyses due to the subsequent elimination of their indicators in the next steps of the index development. The first component of the final PCA explained 51.73% of the total variance in the data. Table 1 shows the descriptive statistics of the 17 variables composing the deprivation index. These indicators showed wide variation between the study areas. For example, literacy rates ranged from 72.99% to 97.63%, internet access ranged from 2.75% to 44.48%, not overcrowded dwellings ranged from 31.97% to 92.28%, the population with private medical insurance ranged from 1.97% to 27.46%, and the population who self-identified as white or mestizo ranged from 4.08% to 99.45%. These indicators showed differences in their relative contribution to the index scores. Piped dwellings and internet access made the highest contributions to the index scores (9.32% and 8.89%, respectively), whereas the proportion of ethnic minorities contributed only 3.87%.

**Table 1:** Descriptive statistics of the 17 indicators used for the construction of the index.

Domains	Indicator	Minimum	Median	Maximum	Contribution (%)*
1. Education	1. Literacy.	72.99	89.75	97.63	6.60
	2. Educational attainment.	14.37	39.23	76.82	5.99
2. Information and Communication	3. Mobile phone access.	4.93	40.57	66.54	8.44
	4. Internet access.	2.75	11.59	44.48	8.89
3. Housing and Urban Environment	5. Main cooking fuel.	9.23	88.22	98.39	4.61
	6. Toilet for household use only.	25.07	75.31	94.27	3.58
	7. Water supply.	10.05	58.68	96.03	7.31
	8. Rubbish collection.	5.90	58.79	98.47	7.10
	9. Toilet to sewer.	0.52	32.17	90.91	6.88
	10. Electricity supply.	26.33	92.21	99.58	5.64
	11. Dwelling overcrowding.	31.97	75.78	92,28	6.05
	12. Pipes into residence/dwelling.	5.07	43.98	84.08	9.32
	13. Compound indicator of dwelling quality: walls, floor, ceiling.	7.55	20.17	57.81	6.49
4. Work and Social Security	14. Private insurance.	1.97	4.51	27.46	4.75
	15. Women’s unpaid working hours ratio.	38.49	44.76	52.80	1.49
	16. Poverty perception.	4.54	7.97	60.31	2.97
5. Historical Discrimination	17. Ethnicity.	4.08	80.96	99.45	3.87

* Contribution, in percentage, of each variable in the construction of the index.

As observed in Table 2, a negative and statistically significant correlation was observed between all selected indicators and the index, except for paid work hours in women, in which higher index scores represent more deprivation and higher variable scores represent less deprivation. The correlations between material deprivation indicators and the index were consistently higher than those observed for the variable related to historical discrimination (ethnicity). The variable most closely correlated with the deprivation index was the presence of pipes in the dwelling (*r* = -0.91).

**Table 2:** Correlation coefficients between socioeconomic indicators used for the construction of the index.

Correlation	1	2	3	4	5	6	7	8	9	10	11	12	13	14	15	16	17	Index
1	1.00	0.73	0.66	0.62	0.47	0.27	0.61	0.64	0.57	0.42	0.45	0.64	0.55	0.46	-0.09	0.29	0.56	-0.76
2	0.73	1.00	0.62	0.76	0.42	0.22	0.49	0.69	0.56	0.19	0.29	0.60	0.76	0.70	0.01	0.25	0.14	-0.73
3	0.66	0.62	1.00	0.72	0.66	0.59	0.54	0.65	0.56	0.70	0.61	0.71	0.61	0.60	-0.12	0.43	0.53	-0.86
4	0.62	0.76	0.72	1.00	0.38	0.34	0.69	0.64	0.77	0.44	0.57	0.76	0.85	0.70	-0.44	0.55	0.34	-0.88
5	0.47	0.42	0.66	0.38	1.00	0.47	0.42	0.70	0.33	0.65	0.42	0.57	0.31	0.36	0.07	0.15	0.34	-0.64
6	0.27	0.22	0.59	0.34	0.47	1.00	0.33	0.42	0.25	0.69	0.62	0.46	0.27	0.30	-0.02	0.22	0.41	-0.56
7	0.61	0.49	0.54	0.69	0.42	0.33	1.00	0.64	0.73	0.59	0.57	0.83	0.50	0.35	-0.43	0.36	0.49	-0.80
8	0.64	0.69	0.65	0.64	0.70	0.42	0.64	1.00	0.58	0.50	0.37	0.72	0.55	0.53	-0.05	0.25	0.37	-0.79
9	0.57	0.56	0.56	0.77	0.33	0.25	0.73	0.58	1.00	0.45	0.61	0.78	0.57	0.39	-0.45	0.24	0.42	-0.78
10	0.42	0.19	0.70	0.44	0.65	0.69	0.59	0.50	0.45	1.00	0.68	0.66	0.23	0.27	-0.20	0.29	0.63	-0.70
11	0.45	0.29	0.61	0.57	0.42	0.62	0.57	0.37	0.61	0.68	1.00	0.65	0.43	0.27	-0.46	0.34	0.61	-0.73
12	0.64	0.60	0.71	0.76	0.57	0.46	0.83	0.72	0.78	0.66	0.65	1.00	0.66	0.47	-0.37	0.39	0.52	-0.91
13	0.55	0.76	0.61	0.85	0.31	0.27	0.50	0.55	0.57	0.23	0.43	0.66	1.00	0.71	-0.34	0.54	0.16	-0.76
14	0.46	0.70	0.60	0.70	0.36	0.30	0.35	0.53	0.39	0.27	0.27	0.47	0.71	1.00	-0.05	0.40	0.14	-0.65
15	-0.09	0.01	-0.12	-0.44	0.07	-0.02	-0.43	-0.05	-0.45	-0.20	-0.46	-0.37	-0.34	-0.05	1.00	-0.60	-0.27	0.36
16	0.29	0.25	0.43	0.55	0.15	0.22	0.36	0.25	0.24	0.29	0.34	0.39	0.54	0.40	-0.60	1.00	0.25	-0.51
17	0.56	0.14	0.53	0.34	0.34	0.41	0.49	0.37	0.42	0.63	0.61	0.52	0.16	0.14	-0.27	0.25	1.00	-0.58

Indicators: 1. literacy; 2. educational attainment; 3. mobile phone access; 4. internet access; 5. main cooking fuel; 6. toilet for household use only; 7. water supply; 8. rubbish collection; 9. toilet to sewer; 10. electricity supply; 11. dwelling overcrowding; 12. pipes into residence/dwelling; 13. compound indicator of dwelling quality: walls, floor, ceiling; 14. private insurance; 15. women’s unpaid working hours ratio; 16. poverty perception; 17. ethnicity.

The final index scores were highly correlated with those obtained from the first PCA with 36 variables (rho = 0.98, p > 0.001).

**Figure 1:**
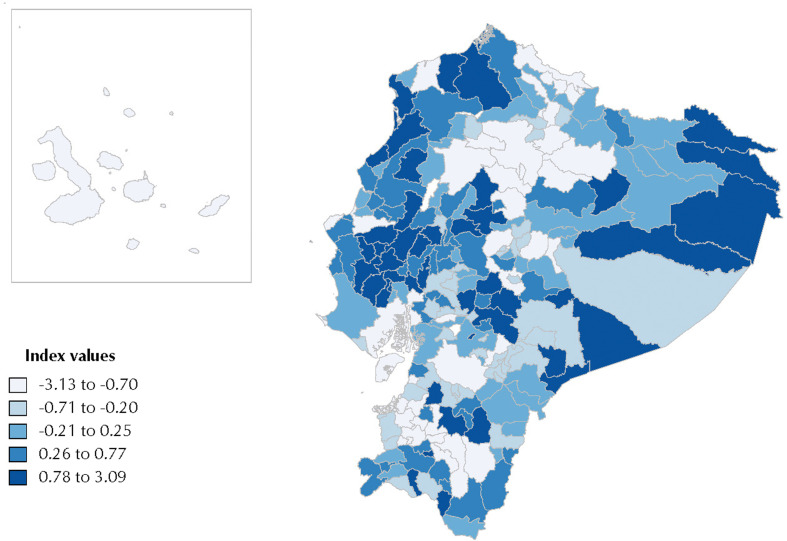
Deprivation Index Scores – Geographical Representation in Quintiles of Deprivation (higher values indicate more deprivation).

**Supplementary Figure 2:**
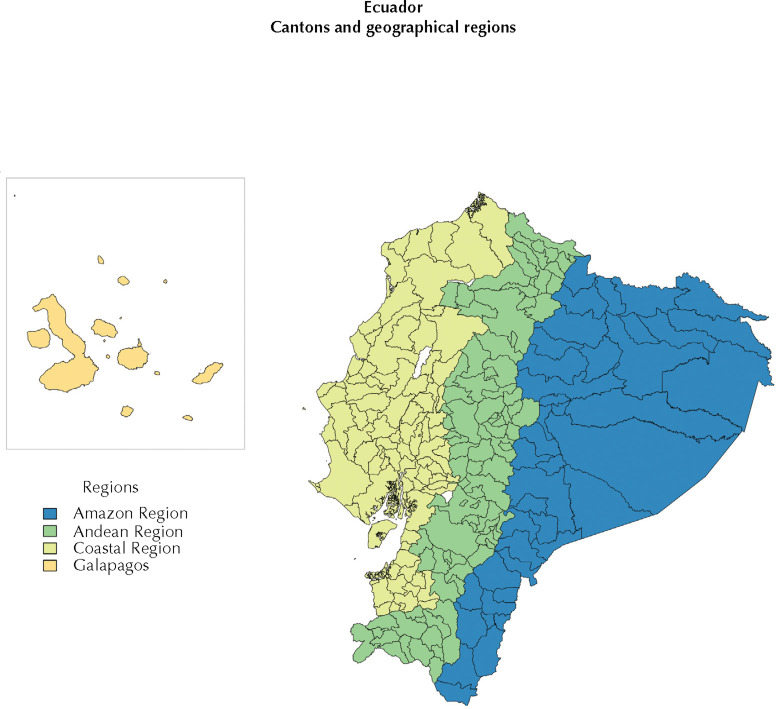
Deprivation Index scores ranking of the 221 cantons of Ecuador across geographical regions.

The index values were unequally distributed among the cantons of Ecuador. Figure 1 shows a geographical pattern, with the most deprived cantons located in the Amazon, central Andean, and northern and central coastal regions. Taisha, located in the Amazon region, is the most deprived canton, with an index score of 3.09; while Rumiñahui, located in the Andean region bordering Quito, is the least deprived canton, with an index score of -3.13 (Supplementary Figure 2). The estimated Moran’s i showed a spatial autocorrelation (i = 0.30, p < 0.001).

### Use of the Index for Public Health Purposes

**Figure 2:**
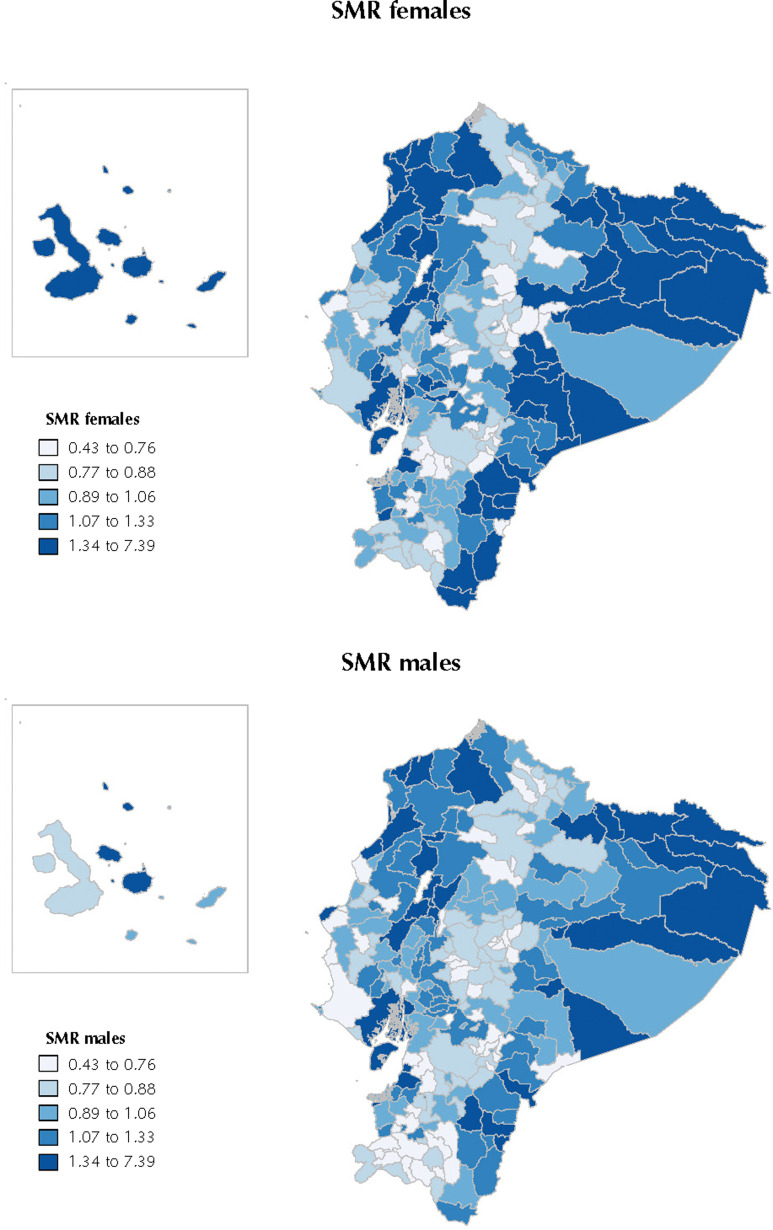
Standardised mortality ratios (SMR) of the cantons by sex.

Figure 2 shows the geographical patterns of SMR for men and women in Ecuador (2009–2011). These patterns were similar to the one observed for the index. The Poisson models fitted for this purpose showed that, SMR increased with higher deprivation levels in both women and men. In women, the relative risk (RR) for each standard deviation increment in the index was 1.06 (95%CI 1.06–1.07). In men, the RR for each standard deviation increase in the index was 1.04 (95%CI 1.03–1.04).

## DISCUSSION

This study shows a deprivation index design for the cantons of Ecuador that captures several deprivation dimensions. The index is composed of 17 variables from public sources. The final index includes indicators related to multiple forms of oppression in a middle-income country with a colonial history such as Ecuador. A geographical pattern of deprivation is evident in the country. The index is positively associated with all-cause mortality in the study areas, demonstrating its potential utility for public health surveillance, studies and public policy planning.

Multidimensional indexes can describe a complex phenomenon such as deprivation with detail and insight. Moreover, the creation of multiple deprivation measures is important to enhance studies on geographical inequalities in health in low- and middle-income countries. In our study, as in other studies that have constructed deprivation indexes in other contexts^26^, material deprivation indicators such as education, work and housing were prioritised by the analyses. Furthermore, it is important to observe the relatively large contribution to the index of indicators that have been considered more sensitive in detecting deprivation in low- and middle-income countries such as the presence of pipes in the dwelling, literacy or water supply^6^.

Our index also examines the contribution of other possible sources of deprivation in the Ecuadorian context. The relative contribution to the index of indicators of access to mobile phones and particularly to the internet may help to explain an emerging digital divide in the country. Consequently, it is possible that “offline” groups may be marginalised from new forms of political, social and economic participation, thereby reproducing or even accelerating social inequalities^14^. Also, the indicator of poverty perception was retained in the construction of the index. In this regard, subjective well-being measures have proved to be associated with a range of health outcomes in high, low- and middle-income countries^29^. This emphasises the importance of developing a specific methodology to construct deprivation indexes in Latin American contexts.

Although ethnicity is a strong social determinant of health^30^ and has been related to socioeconomic inequalities in Ecuador^1^, it showed an unexpected low correlation and contribution to the index, as the geographical pattern of deprivation is similar to the pattern of historically oppressed ethnic groups. A possible explanation is that data were measured by self-identification based on predetermined categories (Ecuadorian census). Possibly, historical discrimination based on race and ethnicity may have resulted in strong pressure to identify with majority or dominant groups (white or mestizo) rather than minority status groups^31^.

Women in unpaid work showed a different direction in the correlation with the deprivation index and made the lowest contribution to its construction. Nevertheless, we retained this indicator based on the conceptual premise that it may provide information on gender inequalities based on the sexual division of work. The unequal use of time between men and women in Ecuador could affect health, as in other countries^18^. Women in Ecuador devote considerably more unpaid hours than men to housework, child and elder care, making them dependent in the income of their partner or family^32^.

The overall geographical pattern of deprivation observed in Ecuador is highly consistent with the distribution of geographical inequalities in historically discriminated areas and oppressed social groups in the country. This pattern is clearly observed in the Amazon, central Andean highlands and north-central coastal regions of Ecuador, for example, which are predominantly inhabited by indigenous, Afro-Ecuadorian and Montubio^1^, historically discriminated ethnical groups.

We used SMR in Ecuador (2009–2011) as a common indicator of health inequalities to illustrate the potential uses of the index in public health. We found that the most deprived cantons were more likely to have higher SMR, showing a gradient. This gradient has been extensively observed in most studies in different countries and settings, but mainly in high-income countries^33^^,^^34^.

This study has some limitations. First, the index is based on data from the Census and Living Conditions Survey. Although these sources provide many interesting indicators, important information that could be relevant to describe material deprivation such as work and labour conditions, and access to public services are not available in these sources. In addition, the information from the Living Conditions Survey is only representative at the provincial level. This could mask inequalities between cantons and could thus be responsible for the minor contribution of LCS variables to the final index. Another limitation could be derived from the characteristics of the study areas. Although cantons are the smallest area, in which the index could be estimated, they are still widely heterogeneous in size and population. We believe that, in the future, the National Statistics Institute could collect and disseminate information in more homogeneous and stable areas. Nevertheless, we also believe that the index covers relevant deprivation dimensions, and that using the information from these relevant sources makes it easier to replicate our results and estimate the evolution of deprivation when these periodical sources of information are updated in Ecuador and similar middle-income countries. Our index is, to the extent of our knowledge, the first multiple deprivation measure constructed for public health purposes at a second administrative level in Ecuador. The development of this kind of new social measure is vital for identifying and understanding the role of the context in diverse health outcomes and health inequalities. Moreover, its development can be of paramount importance for local stakeholders that aim to design and implement policies and to allocate material and social resources to tackle the determinants of health inequalities.

### Conclusions and Implications

In this study, we constructed a deprivation index to identify disadvantaged areas in Ecuador. From a conceptual perspective, the Ecuadorian index incorporates novel indicators to cover wider aspects of deprivation in the current Latin American context. The index scores could be understood as a representation of multiple oppressions reflected in space. Thus, this measurement allows us to analyse the diverse health effects of contextual multiple injustices at the cantonal level in Ecuador. Consequently, this index can be used for health planning and resource allocation. In addition, it allows policy makers and public health professionals to target strategically vulnerabilised populations, and to prioritise interventions and policies adapted to local needs. For researchers, it is a valuable measure to study socioeconomic inequalities using highly diverse health outcomes, including mortality (all-cause and specific causes related to deprivation), morbidity, and access to healthcare”. Finally, we believe that both the methodological and conceptual framework could be useful for future studies in other middle-income countries in Latin America with similar historical, political and social backgrounds.
